# Transactions

**Published:** 1883-02

**Authors:** 


### Transactions.

The volume of Transactions of the last annual meeting of the American Dental Association, has just come to hand. It is well gotten up, both in matter and dress. The publication committee has been rather more prompt than usual, and the work was never better done. This is a valuable volume and should be in the hands of every dentist. It can be obtained of the Secretary, Dr. Geo. H. Cushing, of Chicago.

A large part of this number of the REGISTER is devoted to the publication of the remaining portion of Mr. Mummary's paper, " *On the Relation which Dental Caries—as discovered among the Ancient Inhabitants of Britain, and among existing Aboriginal Races —may be supposed to hold to their food and Social Condition.* Such an interest was shown in the part published in the January number, that it was thought best that the rest should appear unbroken in this. The subject is one of great interest, and as printed hêre may be studied with much profit.

THE following applicants for certificates of qualification, to practice Dentistry in accordance with the laws of Ohio, passed a satisfactory examination before the Ohio State Board of Dental Examiners, at its annual session, held at Columbus, Ohio, December 6th, 7th, and 8th, 1882: William T. Jackman, and Frank A. Sabin, Columbiana, Columbiana County; Will L. Gares, Columbus, Franklin County ; John W. Shults, VanWert, Van-Wert County.

F. H. REHWINKEL,
Chillicothe, February 2, 1883. Sec'y and Treas. of Board.

